# Case Report: Possible causes and management decision-making for near-180° fold-back kinking of a VA-ECMO venous drainage cannula

**DOI:** 10.3389/fcvm.2026.1925357

**Published:** 2026-08-20

**Authors:** Yang Hu, Shujian Zhang, Wei Zhang, Bingjie Lai, Ming Gu

**Affiliations:** 1 Department of Emergency and Critical Care Medicine, Second Hospital of Jilin University, Changchun, Jilin, China; 2 Health Screening Section, Changchun Children’s Hospital, Changchun, Jilin, China

**Keywords:** cannula deformation, cannula kinking, case report, ECMO, extracorporeal membrane oxygenation

## Abstract

**Background:**

Intravascular bending, kinking, or fold-back deformation of an extracorporeal membrane oxygenation (ECMO) venous drainage cannula is a rare cannula-related event. Reports of such events are limited, and no mature or standardized management strategy has been established. We report a case of near-180° fold-back kinking of a venous drainage cannula in the inferior vena cava during venoarterial extracorporeal membrane oxygenation (VA-ECMO) support, aiming to highlight the possibility of cannula deformation during cannulation and to provide a reference for individualized management of similar events.

**Case presentation:**

A 72-year-old woman with non-ST-segment elevation myocardial infarction complicated by refractory cardiogenic shock underwent right femoral venoarterial extracorporeal membrane oxygenation (VA-ECMO) support. After cannulation, bedside ultrasonography and radiography revealed near-180° fold-back kinking of the venous drainage cannula in the inferior vena cava. Because ECMO function remained stable and there was no evidence of vascular injury, bleeding, or thrombotic complications, the clinical team chose conservative observation under close monitoring. Seven days later, the patient was successfully weaned from VA-ECMO. Decannulation was uneventful, and no definite related adverse events were observed before discharge.

**Conclusion:**

During the placement of large-bore ECMO cannulas, premature withdrawal of the guidewire and obturator should be carefully avoided to prevent inadequate cannula support and subsequent deviation or fold-back kinking. For patients with fold-back kinking but stable cannula function and no evidence of vascular injury, bleeding, or thrombosis, conservative observation under close monitoring may be an individualized management option.

## Introduction

Extracorporeal membrane oxygenation (ECMO) is an important form of extracorporeal life support for critically ill patients, and the stability of vascular access directly affects the effectiveness of clinical support (1–5). Malposition of the venous drainage cannula is a relatively common cannula-related problem during ECMO support. Previous reports have described inadvertent placement of the drainage cannula into the hepatic vein, azygos vein, or other abnormal venous branches, which may lead to impaired venous drainage, reduced ECMO blood flow, or local vascular injury (5–17). For definite cannula malposition, repositioning or cannula replacement is usually performed to restore effective drainage (5, 10, 14–16).

Unlike the above-mentioned cannula malposition, obvious bending, fold-back kinking, or deformation of the venous drainage cannula within the intended vascular pathway has rarely been reported, and clinical awareness and management experience remain limited. In particular, near-180° fold-back kinking of the cannula in the inferior vena cava has been scarcely described. Herein, we report a rare case of near-180° fold-back kinking of a venoarterial extracorporeal membrane oxygenation (VA-ECMO) venous drainage cannula in the inferior vena cava, aiming to deepen clinical understanding of cannula deformation during cannulation and to provide a reference for individualized management of similar events.

## Case presentation

A 72-year-old woman with non-ST-segment elevation myocardial infarction complicated by refractory cardiogenic shock underwent VA-ECMO support. A 21-Fr venous drainage cannula was inserted through the right femoral vein, with a planned insertion depth of approximately 45 cm. Before the venous drainage cannula had reached its intended position, the guidewire and inner stylet were removed, after which the cannula was advanced further without internal support to a final insertion depth of approximately 45 cm. The arterial return cannula was inserted through the right femoral artery, with a size of 15 Fr and an insertion depth of approximately 17 cm. After VA-ECMO initiation, the patient's circulatory status improved, and her hemodynamics gradually stabilized.

**Figure 1:**
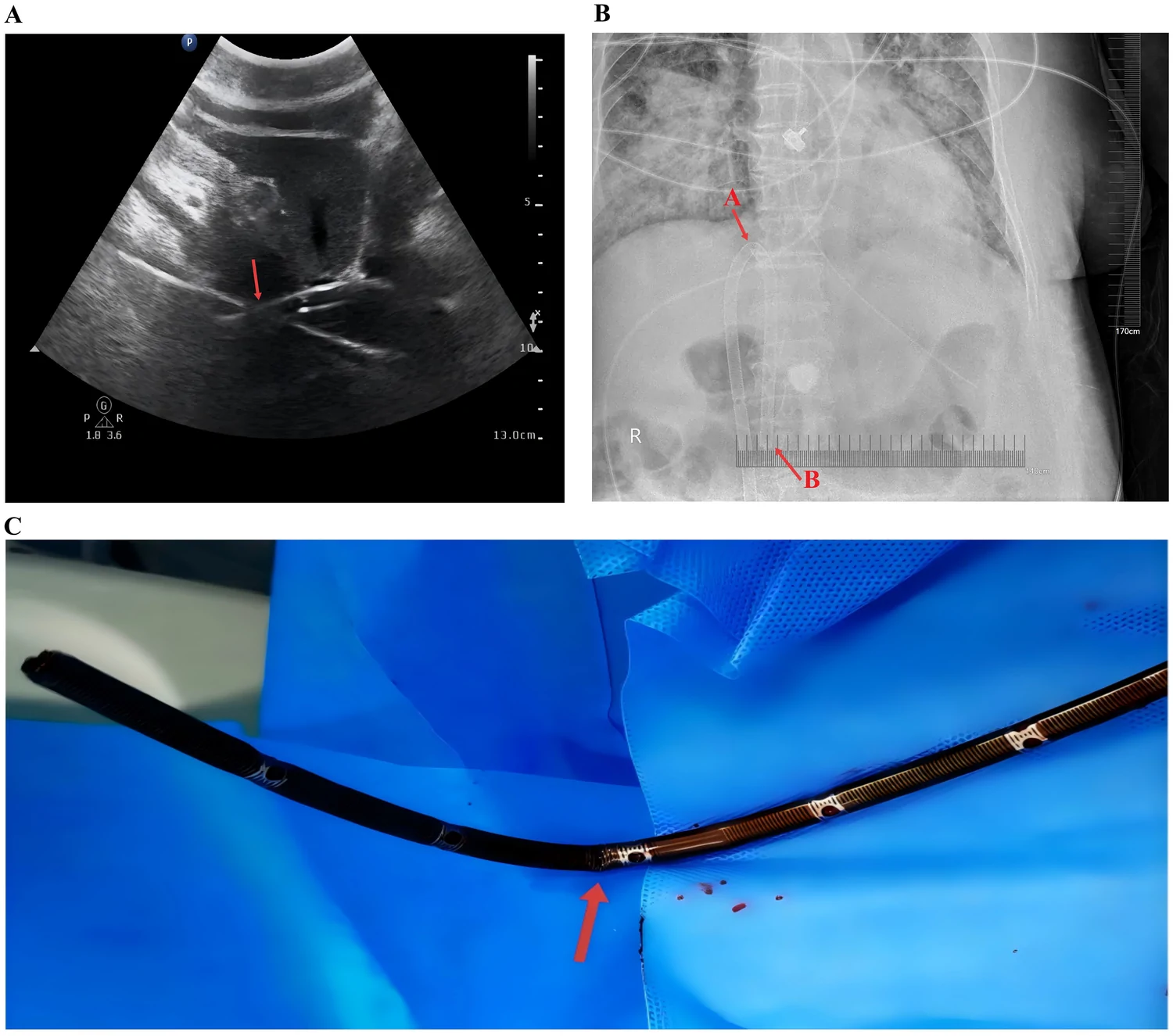
Imaging findings and post-removal appearance of the fold-back kink of the VA-ECMO venous drainage cannula. **(A)** Bedside ultrasonography showed that the ECMO venous drainage cannula remained within the vascular lumen, with an obvious focal bending configuration. The arrow indicates the kinked segment of the cannula. **(B)** Bedside radiography showed a marked fold-back kink of the ECMO venous drainage cannula inserted through the femoral vein along the course of the IVC. Arrow A indicates the site of cannula bending/fold-back deformation, and arrow B indicates the position of the cannula tip. **(C)** Appearance of the venous drainage cannula after VA-ECMO decannulation. The arrow indicates persistent bending of the cannula. The cannula remained structurally intact, with no fracture or visible damage.

Following ECMO cannulation, routine bedside ultrasonography was performed to evaluate the position and drainage function of the venous drainage cannula. During the examination, although the cannula remained within the lumen of the inferior vena cava, a marked focal abnormal bend was identified (Figure 1A). Subsequent bedside radiography further demonstrated near-180° fold-back kinking of the cannula along the course of the inferior vena cava, with the cannula tip directed back toward the femoral vein (Figure 1B). Despite the marked imaging abnormality, ECMO blood flow remained stable. Follow-up bedside ultrasonography and imaging showed no evidence of extravascular cannula migration, local hematoma, intraperitoneal bleeding, retroperitoneal bleeding, or other signs of vascular injury.

After comprehensive assessment of cannula function, circulatory support, evidence of complications, and the risks of recannulation, the clinical team did not immediately reposition or replace the cannula. Instead, a conservative management strategy under close monitoring was adopted, including continuous observation of ECMO parameters, serial bedside ultrasonography and radiography, and active screening for complications such as bleeding, thrombosis, hemolysis, and impaired lower-limb venous return. After 7 days of VA-ECMO support, the patient's cardiac function gradually recovered, and she met the criteria for weaning. Given the near-180° fold-back configuration of the venous drainage cannula, direct decannulation was considered to carry a potential risk of traction injury to the venous wall, venous intimal damage, or bleeding. Therefore, the clinical team elected to perform decannulation in the operating room with the vascular surgery team on standby to allow immediate management should unexpected vascular complications occur.

The entire decannulation procedure was performed under real-time ultrasonographic guidance. The cannula was withdrawn slowly and incrementally, with only a few centimeters removed at each step. After each withdrawal, ultrasonography was repeated to evaluate the cannula configuration, its relationship to the venous wall, and the presence of any newly developed perivascular fluid collection or other signs of vascular injury. No guidewire or other adjunctive maneuvers were used during the decannulation process. Ultimately, the cannula was removed completely without significant resistance. No active bleeding, significant hemodynamic instability, or ultrasonographic evidence of vascular injury was observed during or after decannulation. Gross inspection of the removed cannula demonstrated persistence of the folded configuration, while the cannula remained structurally intact without fracture or visible damage (Figure 1C).

## Discussion

Obvious bending, kinking, or fold-back deformation of an ECMO venous drainage cannula is a rare cannula-related event. Near-180° fold-back kinking of the cannula in the inferior vena cava is even more uncommon. Previously reported cannula malposition may not only impair venous drainage, resulting in reduced effective ECMO blood flow, increased recirculation, and inadequate oxygen delivery, but also predispose patients to serious vascular complications. Persistent compression of the venous wall by the malpositioned cannula or mechanical traction during cannula manipulation may further lead to venous injury, vascular perforation, retroperitoneal or intraperitoneal hemorrhage, and even life-threatening complications (5–17). Representative reports of ECMO venous cannula malposition and associated complications are summarized in Table 1. In contrast, the distinctive feature of the present case is that the venous drainage cannula did not completely enter an incorrect vessel but instead developed marked fold-back kinking within the intended venous pathway. Despite the obvious imaging abnormality, ECMO blood flow, drainage status, and circulatory support remained stable, and no vascular injury, bleeding, or thrombotic complications were observed. Therefore, the value of this case lies not only in reporting a rare cannula deformation, but also in emphasizing that cannula function and the overall patient risk should be incorporated into clinical decision-making when similar situations are encountered.

**Table 1:** Reported ECMO venous cannula malpositions involving specific vascular structures.

Reference	Misplaced vessel/anatomical structure	Major complication	Management
Balmaks et al., 2013 (5)	Azygos vein	Inadequate venous drainage	Cannula repositioning
Choi et al., 2016 (16)	Azygos vein	Reduced ECMO flow	Cannula repositioning
Mayer et al., 2019 (6)	Azygos vein (interrupted IVC with azygos continuation)	Cannula malposition	Individualized management
Winiszewski et al., 2018 (7)	Accessory hepatic vein	Hepatic vascular injury	Ultrasound-guided repositioning
Yastrebov et al., 2013 (14)	Hepatic vein (return jet directed into the hepatic vein)	Severe recirculation	Cannula repositioning
Li et al., 2025 (10)	Middle hepatic vein	Inadequate venous drainage	Ultrasound-guided repositioning
Tominaga et al., 2018 (15)	Ascending lumbar vein	Retroperitoneal hemorrhage	Cannula repositioning
Honda et al., 2024 (17)	Ascending lumbar vein	Risk of vascular injury during removal	Balloon-assisted staged removal
Salamo et al., 2024 (8)	Lumbar vein	Inferior vena cava perforation	Image-guided removal
Runkel et al., 2019 (11)	Azygos vein	Cannula malposition; however, adequate venous drainage was maintained due to the anomalous venous anatomy	Individualized management
Hirose et al., 2012 (9)	Right ventricle	Cardiac perforation and tamponade	Emergency surgery
De Cassai et al., 2025 (12)	Extravascular intraperitoneal placement	Massive intra-abdominal hemorrhage	Surgical removal
Ozawa et al., 2024 (13)	Ascending lumbar vein	Common iliac vein perforation and liver injury	Laparotomy with surgical cannula removal and iliac vein repair

In the present case, the abnormal bending of the venous drainage cannula was identified during routine bedside ultrasonography performed immediately after cannulation, suggesting that the deformity was more likely to have occurred during the cannulation procedure rather than during subsequent ECMO support. However, the exact mechanism underlying the near-180° fold-back of the venous drainage cannula remains uncertain. Several possible mechanisms may account for this phenomenon. First, during femoral venous cannulation, the guidewire or cannula may deviate from the intended inferior vena cava pathway and enter the contralateral iliac–femoral venous system or a hepatic vein. When the guidewire or cannula crosses the iliocaval confluence and enters the contralateral common iliac, external iliac, or femoral vein, the distal cannula may be redirected caudally rather than advancing cranially within the inferior vena cava. Similarly, inadvertent entry into a hepatic vein may create focal angulation and constrain further advancement at the junction between the hepatic vein and the inferior vena cava. In either situation, the direction of advancement, the point of contact with the vessel wall, and the distribution of mechanical stress may change substantially. If the distal segment becomes constrained while the proximal cannula continues to be advanced, the axial advancement force may be converted into a local bending force, resulting in mechanical buckling and eventual fold-back of the cannula. Entry into the lumbar vein or another collateral venous branch may produce a similar mechanism. Second, when the cannula tip is unable to advance further because of venous anatomy, vascular tortuosity, or increased local resistance, continued advancement of the proximal cannula may result in mechanical buckling, ultimately leading to a near-180° fold-back configuration. Finally, external factors after cannulation, such as patient repositioning, traction from the ECMO circuit, or inadequate cannula fixation, may also contribute to secondary cannula deformation.

Based on the specific cannulation procedure in this case, we speculate that the nearly 180° fold-back of the venous drainage cannula may have resulted from the combined effects of premature removal of the guidewire and inner stylet and subsequent deviation of the cannula into a non-target vein or venous branch. After removal of the guidewire and inner stylet, the cannula lost the internal axial support required to maintain its structural stability, resulting in reduced resistance to buckling and increased flexibility. Continued advancement under these conditions may have rendered the cannula more susceptible to bending and deviation from its intended course. However, the loss of internal support alone would primarily increase the tendency of the cannula to bend or buckle, whereas deviation into a non-target vein or venous branch may have provided the additional anatomical and mechanical conditions required for the development of a nearly 180° fold-back configuration. When an inadequately supported cannula enters the contralateral iliac vein, hepatic vein, lumbar vein, or another collateral venous branch, its direction of advancement, point of contact with the vessel wall, and site and direction of force application may change substantially. Because of the reduced rigidity and axial support of the cannula, even if its tip encountered local resistance, the resulting change in resistance may have been subtle or gradually progressive rather than abrupt and pronounced, and therefore may not have produced sufficiently distinct tactile feedback to be readily recognized by the operator during cannulation. As the cannula continued to advance along an abnormal pathway, its distal segment may have been constrained by the course of the venous branch or by contact with the vessel wall, while the proximal portion continued to move forward. The resulting axial force may therefore have been converted into a local bending force, altering the direction and distribution of mechanical stress on the cannula and leading to progressive mechanical buckling and, ultimately, a nearly 180° fold-back. Therefore, we consider that the cannula fold-back in this case was unlikely to have been caused by a single factor, but rather by the interaction between insufficient internal support caused by premature removal of the guidewire and inner stylet and an abnormal cannula trajectory caused by possible entry into a non-target vein or venous branch. Although this proposed mechanism appears to be most consistent with the procedural course and imaging findings in this case, the specific venous pathway entered by the cannula and the exact process leading to its fold-back cannot be definitively established because continuous real-time imaging was not available during cannulation.

For this type of cannula deformation, management decisions should not be based solely on the degree of kinking observed on imaging. Instead, cannula function, the effectiveness of ECMO support, evidence of complications, and the risks of further intervention should be comprehensively evaluated. Previous studies have shown that peripheral ECMO cannulation may be associated with vascular injury, bleeding, thrombosis, limb ischemia, and cannula-related mechanical complications (18). In addition, a systematic review and meta-analysis including 159 studies and 21,942 adult patients receiving VA-ECMO reported that the incidence of major bleeding was 40% (95% CI, 36%–44%), and that of major thrombotic complications was 17% (95% CI, 14%–19%) (19). Therefore, when marked fold-back kinking is present but cannula function and circulatory support remain stable, and there is no evidence of bleeding, thrombosis, worsening hemolysis, or vascular injury, immediate cannula repositioning or replacement may not be the only option. In this case, because the patient was still receiving VA-ECMO support and anticoagulation, further cannula adjustment or recannulation could have introduced additional risks of vascular injury, bleeding, and hemodynamic instability. Considering these factors, a conservative management strategy was adopted, consisting of serial bedside ultrasonography and radiography to assess changes in cannula position, continuous monitoring of ECMO operating parameters, and systematic surveillance for cannula-related complications. Low ECMO flow, circuit chattering, drainage insufficiency, deterioration of circulatory or oxygenation support, worsening hemolysis, thrombosis, or evidence of vascular injury may indicate that the cannula deformity has begun to compromise ECMO performance and should prompt escalation of management (20, 21). During conservative observation, repeat imaging should be performed according to the patient's clinical condition and ECMO performance, typically within several hours after detection of the abnormality and again within 24 h, with subsequent monitoring intervals individualized according to clinical stability. In the absence of these high-risk findings, close observation may be continued. However, if any of these abnormalities develop, the cannula position should be reassessed promptly, and cannula repositioning, recannulation, or replacement should be considered. The proposed monitoring intervals and escalation criteria are intended as practical clinical surveillance parameters rather than universally validated thresholds and should be individualized according to the patient's baseline condition, ECMO mode and circuit configuration, device-specific alarm limits, institutional protocols, and dynamic clinical changes. Because no universally accepted, evidence-based threshold for drainage pressure has been established, intervention decisions should not be based on a single drainage-pressure value alone. In contrast, a plasma-free hemoglobin level >50 mg/dL should prompt further evaluation for clinically significant hemolysis. The proposed monitoring parameters, timelines, and escalation criteria are summarized in Table 2.

**Table 2:** Proposed monitoring schedule and escalation criteria during conservative management of patients with bending or deformation of the ECMO venous drainage cannula.

Assessment category	Monitoring indicators	Suggested monitoring schedule	Proposed escalation criteria
ECMO flow and drainage performance	ECMO blood flow, pump speed, drainage/inlet pressure when available, pre- and post-oxygenator pressures, and transmembrane pressure gradient	Continuous monitoring, with hourly documentation during the first 24 h and every 2–4 h thereafter if stable	A sustained decrease in ECMO flow of ≥10% from the preceding stable baseline for >5 min; recurrent low-flow alarms or circuit chattering; inability to maintain the target flow without increasing pump speed; or a drainage pressure becoming ≥20 mmHg more negative than baseline or reaching the manufacturer-specific alarm limit
Oxygenation and circulation	SpO_2_, PaO_2_, PaCO_2_, MAP, lactate, urine output, and vasoactive drug dose	Continuous monitoring of SpO_2_, MAP, and urine output; arterial blood gas and lactate every 4–6 h during the first 24 h and every 6–12 h thereafter if stable	MAP <65 mmHg; an increase in vasoactive drug dose of ≥20%; an increase in lactate of ≥2 mmol/L or ≥20% from the previous value; or progressive deterioration in oxygenation or tissue perfusion
Hemolysis	Plasma-free hemoglobin, LDH, bilirubin, hemoglobin level, and urine color	Baseline assessment after detection of cannula deformation, every 6–12 h during the first 24 h, and at least once daily thereafter if stable	Plasma-free hemoglobin >50 mg/dL or a rapid upward trend; LDH increase of ≥50% from baseline; unexplained hemoglobin decrease of ≥2 g/dL within 24 h; or newly developed dark urine/hemoglobinuria
Coagulation and thrombosis	Platelet count, D-dimer, fibrinogen, APTT, ACT, anti-factor Xa activity, and ultrasonographic evidence of thrombosis	According to the institutional anticoagulation protocol, generally every 4–6 h until anticoagulation is stable and every 6–12 h thereafter	Newly detected cannula-associated or venous thrombosis; platelet count decrease of ≥30% from baseline without another clear cause; progressive venous outflow obstruction; or inability to maintain the prescribed anticoagulation target
Vascular complications and cannula configuration	Cannula position and configuration, relationship to the venous wall, perivascular fluid collection, retroperitoneal or intraperitoneal fluid, and lower-limb venous return	Bedside ultrasonography immediately after detection, at 2–4 h, 6–12 h, and 24 h, and then every 24 h if stable; radiography immediately after detection and again within 12–24 h, or sooner if ECMO performance or clinical status changes	Progressive cannula displacement or worsening deformation; newly developed perivascular fluid collection or hematoma; evidence of vascular perforation; impaired iliac or femoral venous flow; or new lower-limb swelling

ECMO, extracorporeal membrane oxygenation; SpO_2_, peripheral oxygen saturation; PaO_2_, arterial partial pressure of oxygen; PaCO_2_, arterial partial pressure of carbon dioxide; MAP, mean arterial pressure; LDH, lactate dehydrogenase; APTT, activated partial thromboplastin time; ACT, activated clotting time.

This case report has several limitations. First, this is a single-center, single-case report and therefore cannot demonstrate that all ECMO cannula kinking or deformation can be safely managed conservatively. Second, because the patient was critically ill, CT or angiography was not further performed; therefore, assessment of the local cannula configuration, degree of luminal compression, and stress on the vascular wall was limited. In addition, although no thrombotic adverse events were observed after decannulation in this case, whether cannula kinking increases the subsequent risk of thrombosis, hemolysis, or vascular complications remains uncertain. Further accumulation of cases and long-term follow-up data are needed. Finally, because continuous imaging was not available during the cannulation procedure, the exact mechanism underlying the near-180° fold-back of the venous drainage cannula could not be definitively determined.

## Conclusion

During the placement of large-bore ECMO cannulas, premature withdrawal of the guidewire and obturator should be carefully avoided to prevent inadequate cannula support and subsequent deviation or fold-back kinking. For patients with fold-back kinking but stable cannula function and no evidence of vascular injury, bleeding, or thrombosis, conservative observation under close monitoring may be an individualized management option.

## Data Availability

The original contributions presented in the study are included in the article/Supplementary Material, further inquiries can be directed to the corresponding author.
